# Quality, reliability, and content completeness of Chinese-language short videos on impacted wisdom teeth on TikTok and Bilibili: a cross-sectional study

**DOI:** 10.1186/s12903-026-08553-7

**Published:** 2026-05-11

**Authors:** Feng Cai, Jiayuan Zou, Wending Gao

**Affiliations:** Department of Stomatology, The Central Hospital of Wuhan, Tongji Medical College, Huazhong University of Science and Technology, Wuhan, 430014 China

**Keywords:** Impacted wisdom teeth, Social media, Health communication, Information quality, Short-video platforms

## Abstract

**Background:**

Short-video platforms have become major channels for disseminating public health information. This study evaluated the quality, reliability, and content completeness of videos on impacted wisdom teeth on TikTok and Bilibili. It examined whether platform type, uploader category, and user engagement metrics were associated with these outcomes.

**Methods:**

A cross-sectional analysis was conducted on November 20, 2025. The top 150 videos from each platform were retrieved using the standard Chinese clinical term for impacted wisdom teeth. After screening, 199 videos were included. Quality and reliability were assessed using the Global Quality Score (GQS), modified DISCERN (mDISCERN), and Journal of the American Medical Association (JAMA) benchmark criteria. Content completeness was assessed using an 8-item clinical checklist. Uploaders were categorized as specialized healthcare professionals (SHCPs), non-specialized healthcare professionals (NSHCPs), and individual users (IUs). Non-parametric tests and Spearman’s correlation analysis were used.

**Results:**

Overall video quality and reliability were moderate, with median GQS, mDISCERN, and JAMA scores of 3.00. An “indication-heavy, contraindication-light” pattern was observed: indications (76.38%) and definitions (68.34%) were frequently covered, whereas contraindications (5.53%) were rarely addressed. Videos uploaded by SHCPs had significantly higher quality and reliability scores than those uploaded by IUs (all *P* < 0.001). Engagement metrics, including likes and shares, were not positively correlated with the established quality measures.

**Conclusions:**

Short videos on impacted wisdom teeth on Chinese short-video platforms were of moderate quality and provided limited coverage of contraindications and other risk-related information. Videos uploaded by specialized healthcare professionals tended to provide more reliable and complete information than those from non-professional sources. In contrast, user engagement metrics were not reliable indicators of informational quality. These findings may inform efforts to strengthen evidence-based dental communication on short-video platforms.

**Supplementary Information:**

The online version contains supplementary material available at 10.1186/s12903-026-08553-7.

## Background

 Impacted wisdom teeth represent a prevalent developmental anomaly, frequently leading to clinical sequelae such as pericoronitis, adjacent tooth periodontitis, and odontogenic cysts or tumors [1–4]. Given that surgical extraction is among the most common procedures in oral and maxillofacial surgery [5–7], this condition imposes significant physical and economic burdens globally [8–11]. For patients, access to accurate health information is critical to facilitate informed decision-making, alleviate preoperative anxiety, and ensure adherence to postoperative care protocols [12].

In the digital era, short-video platforms have emerged as dominant channels for disseminating health information, characterized by high accessibility and rapid transmission [13–15]. However, the low-threshold nature of content creation raises substantial concerns regarding information quality [16]. Unlike traditional medical resources, social media content lacks rigorous peer review, increasing the risk of disseminating misleading or incomplete clinical advice [17]. While previous infodemiology studies have evaluated various dental topics on social media [18, 19], a systematic assessment of content specifically related to impacted wisdom teeth, which is a surgical procedure often associated with high patient apprehension and specific postoperative risks, remains limited.

In China, TikTok (known domestically as Douyin) and Bilibili are the two leading platforms, yet they differ significantly in video length, user demographics, and content style [20, 21]. TikTok is characterized by strict duration constraints, a fast-paced aesthetic, and a highly algorithm-driven feed designed for immediate engagement. Conversely, Bilibili caters to a predominantly younger demographic and favors a community-driven environment with longer, more detailed, and often serialized tutorial formats. These distinct platform-specific characteristics, combined with the diverse professional backgrounds of the uploaders, likely influence how dental health information is structured and consumed.

Accordingly, this study evaluated the quality, reliability, and content completeness of short videos on impacted wisdom teeth on TikTok and Bilibili. It examined whether platform type, uploader category, and user engagement metrics were associated with these outcomes.The study hypothesized that videos uploaded by specialized healthcare professionals would provide higher-quality, more complete information than videos from other sources. higher user engagement would not necessarily correspond to higher informational quality.

## Methods

### Data retrieval and collection

A cross-sectional search was conducted on TikTok and Bilibili on November 20, 2025, using the standard Chinese clinical term for impacted wisdom teeth. For each platform, the first 150 videos returned by the default comprehensive ranking were retrieved.

The default comprehensive ranking was used because it represents the standard search output shown to ordinary users and reflects multiple platform-derived signals, including keyword relevance, engagement indicators, upload recency, and account authority. Restricting the sample to the top-ranked results was intended to reflect the information most readily encountered by users in routine searches [22]. However, because rankings are platform-dependent and may change over time, this sampling approach may not capture all relevant videos on either platform.

A single-keyword strategy was adopted because the selected term is the standard Chinese clinical expression for this condition and is widely used in routine written searches. Nevertheless, videos labeled with less formal expressions or other linguistic variants may not have been captured, including colloquial or region-specific expressions. To reduce personalized recommendation bias, all searches were performed using newly registered accounts with no prior search history, and application caches were cleared before data retrieval.

**Fig. 1:**
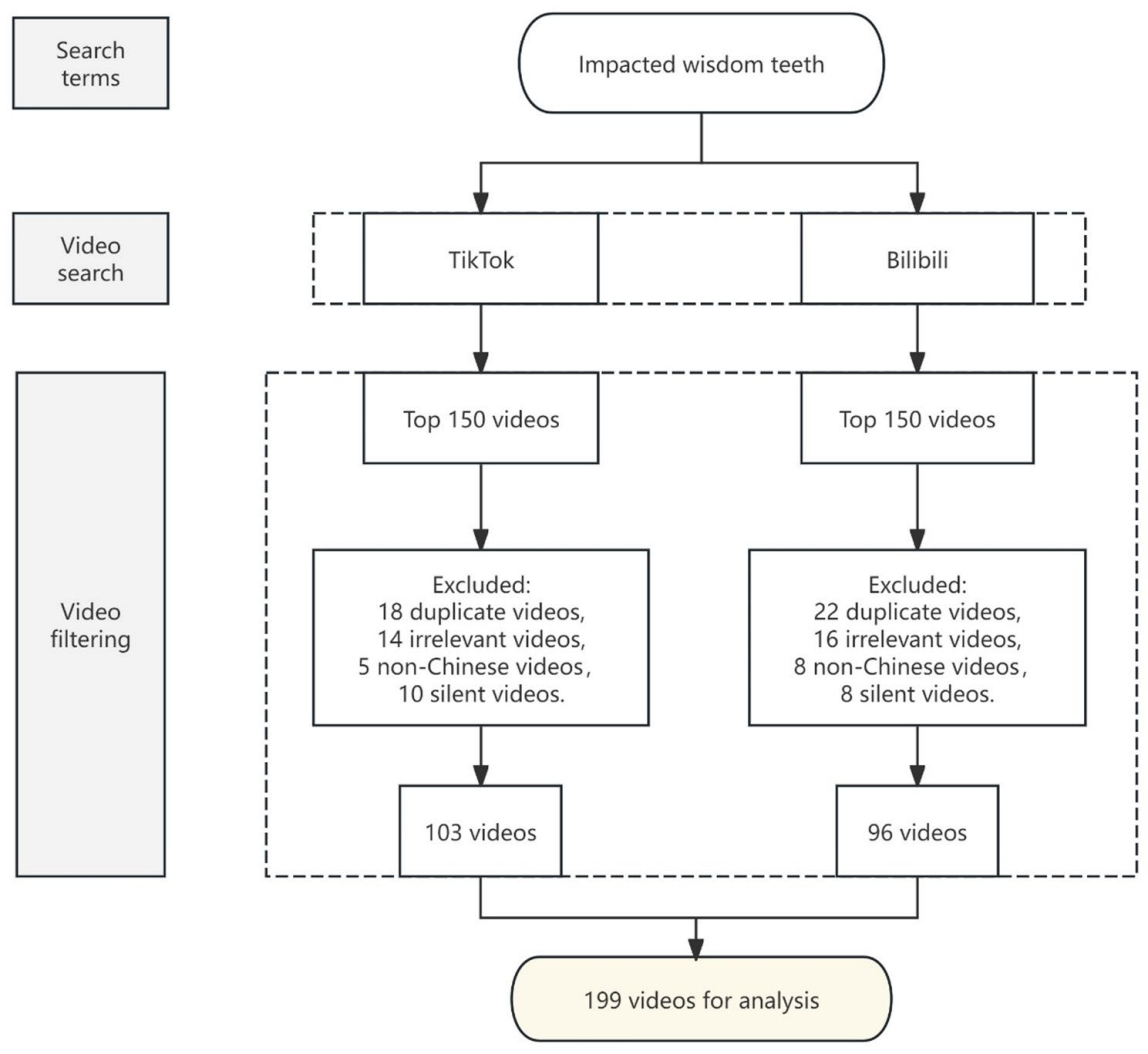
Video selection flowchart. Flowchart showing the search, screening, and selection process for videos on impacted wisdom teeth from TikTok and Bilibili. The standard Chinese clinical search term was used on both platforms. After exclusion of duplicates, irrelevant, non-Chinese, and silent videos, 199 videos were included in the final analysis

Basic video characteristics, including web links, duration, likes, saves, comments, shares, and uploader identity, were recorded on the day of retrieval. Duplicate, irrelevant, non-Chinese, and silent videos were excluded. After screening, 199 videos were included in the final analysis, comprising 103 videos from TikTok and 96 videos from Bilibili (Fig. 1).

Uploaders were classified according to official platform verification into three categories: specialized healthcare professionals (SHCPs; e.g., oral and maxillofacial surgeons), non-specialized healthcare professionals (NSHCPs; e.g., general dentists, physicians, and nurses), and individual users (IUs; e.g., patients and other laypersons). Video assessment was completed between November 22 and 24, 2025.

### Video assessment

Video quality and reliability were assessed using the Global Quality Score (GQS) [23], modified DISCERN (mDISCERN) [24, 25], and the Journal of the American Medical Association (JAMA) benchmark criteria [26]. The scoring details of these instruments are provided in Supplementary Tables S1-S3.

Content completeness was assessed using the Impacted Wisdom Teeth Content Completeness Score (IWT-CCS-8), a study-specific 8-item checklist developed from relevant international literature [27, 28] and Chinese consensus guidance [29–31] on the management of impacted wisdom teeth. Each domain was scored dichotomously (0 = absent; 1 = present), yielding a total score ranging from 0 to 8. The eight domains were definition, indications, contraindications, advantages, procedures, complications, prognosis, and cost (Table 1).

**Table 1:** IWT-CCS-8 scoring domains and criteria

Domain	Description	Criteria for 1 point
Definition	Basic concept	The video explains what an impacted wisdom tooth is, such as failure of normal eruption due to insufficient space, abnormal angulation, or partial or complete coverage by bone or soft tissue.
Indications	Reasons for extraction	The video mentions specific reasons for removal, such as recurrent pericoronitis, caries or damage to adjacent teeth, cystic or periodontal lesions, pain, or orthodontic needs.
Contraindications	Reasons to defer extraction	The video mentions situations in which extraction should be postponed or carefully assessed, such as uncontrolled acute inflammation, poor systemic condition, or absence of a clear surgical indication.
Advantages	Potential benefits of extraction	The video explains potential benefits of removal, such as preventing recurrent infection, protecting the adjacent second molar, or reducing the risk of further pathology.
Procedures	Surgical process	The video describes the main surgical steps, such as local anesthesia, incision or flap elevation, bone removal, tooth sectioning, extraction, and suturing.
Complications	Risks and adverse effects	The video mentions possible complications, such as pain, swelling, bleeding, infection, dry socket, limited mouth opening, or inferior alveolar nerve injury or numbness.
Prognosis	Recovery and aftercare	The video provides postoperative advice or recovery information, such as gauze pressure, cold compress, diet modification, oral hygiene, medication, or follow-up care.
Cost	Financial aspects	The video mentions treatment cost, factors affecting cost, or whether the procedure may be covered by health insurance.

*IWT-CCS-8* Impacted Wisdom Teeth Content Completeness Score

Each domain was scored as 0 (not mentioned) or 1 (mentioned), yielding a total score ranging from 0 to 8

Before the formal assessment, the two primary assessors (JZ and WG) underwent standardized training and completed a consensus session. A pilot calibration exercise was then performed on a random subsample of 20 videos, yielding excellent inter-rater agreement (Cohen’s kappa = 0.84). All included videos were subsequently rated independently by the two assessors. Disagreements were resolved through discussion with a third senior assessor (FC).

### Statistical analysis

Statistical analyses were performed using R software (version 4.3.3). The normality of continuous variables was assessed using the Shapiro-Wilk test. As the data were non-normally distributed, continuous variables are presented as medians with interquartile ranges (IQRs), and categorical variables as frequencies and percentages. Exact non-parametric 95% confidence intervals (CIs) for the medians of the primary outcome scores were calculated using binomial order statistics.

Comparisons between TikTok and Bilibili were performed using the Mann-Whitney U test. Comparisons among uploader categories (SHCPs, NSHCPs, and IUs) were performed using the Kruskal-Wallis test; when the overall test was significant, Dunn’s post-hoc test was used for pairwise comparisons. Spearman’s rank correlation analysis was used to assess associations among video characteristics, engagement metrics, and quality scores. All tests were two-sided, and *P* < 0.05 was considered statistically significant.

## Results

### Baseline video characteristics

The initial search retrieved 300 videos, with 150 from TikTok and 150 from Bilibili. After applying the inclusion and exclusion criteria, 199 videos were included in the final analysis, comprising 103 (51.76%) from TikTok and 96 (48.24%) from Bilibili (Fig. 1).

**Fig. 2:**
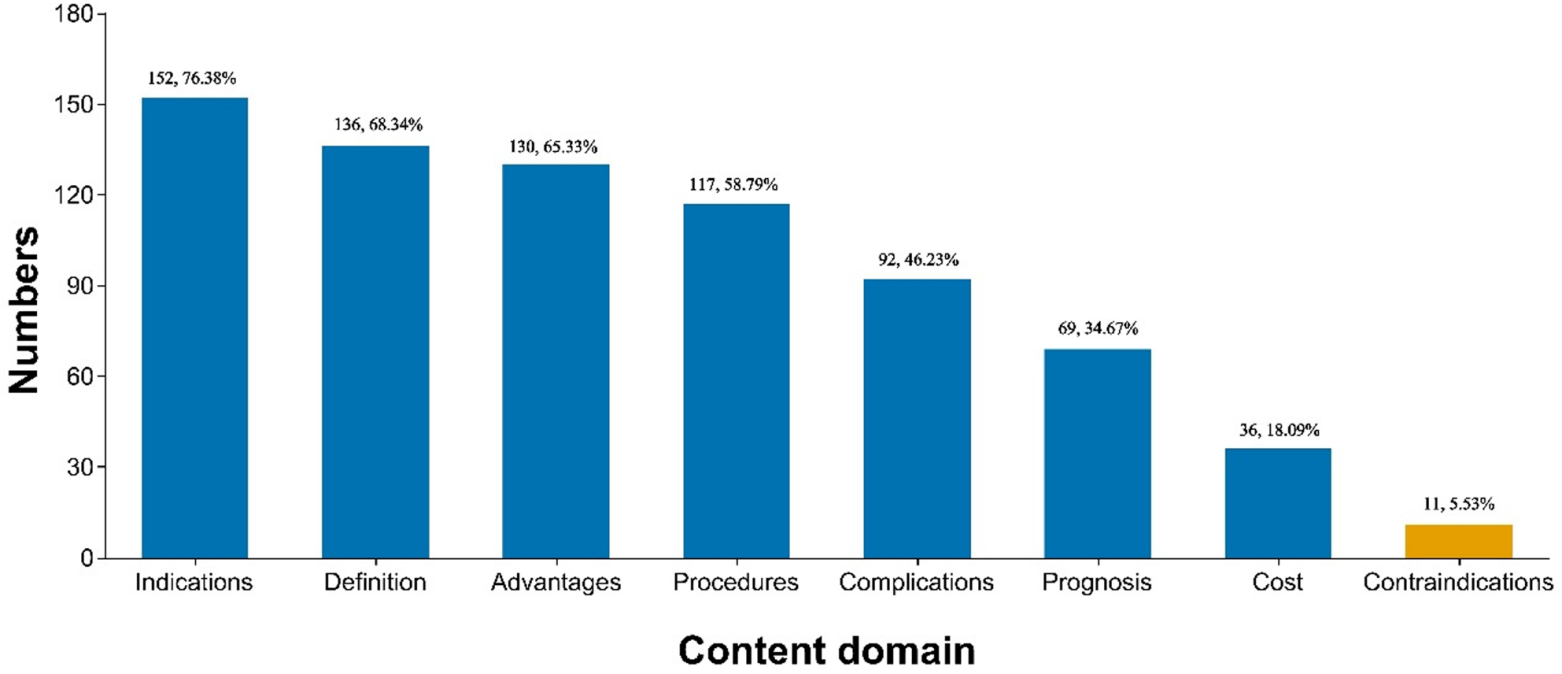
Content domains in included videos. Bar chart showing the number and percentage of included videos that covered each of the eight clinical domains in the IWT-CCS-8: definition, indications, contraindications, advantages, procedures, complications, prognosis, and cost. Data are based on 199 videos

NSHCPs accounted for the largest proportion of uploaders (54.27%), followed by SHCPs (24.62%) and IUs (21.11%). The distribution of content themes is shown in Fig. 2. “Indications” was the most frequently covered domain (76.38%), followed by “Definition” (68.34%) and “Advantages” (65.33%). By contrast, “Contraindications” (5.53%) and “Cost” (18.09%) were infrequently mentioned.

Across the full sample, the median video duration was 137.00 s (IQR: 51.50–317.00). Median engagement metrics were 374.00 likes (IQR: 99.50-1241.50), 102.00 saves (IQR: 39.50-267.50), 133.00 comments (IQR: 35.00-489.00), and 125.00 shares (IQR: 19.00-742.00). The median completeness score was 4.00 (IQR: 3.00–5.00), whereas the median GQS, mDISCERN, and JAMA scores were all 3.00. Detailed baseline characteristics and outcome scores are presented in Table 2.

**Table 2:** Characteristics of included videos

Characteristic	Total (*N* = 199)
Platform, *N* (%)	
TikTok	103 (51.76%)
Bilibili	96 (48.24%)
Content domain, *N* (%)	
Definition	136 (68.34%)
Indications	152 (76.38%)
Contraindications	11 (5.53%)
Advantages	130 (65.33%)
Procedures	117 (58.79%)
Complications	92 (46.23%)
Prognosis	69 (34.67%)
Cost	36 (18.09%)
Uploader category, *N* (%)	
IUs	42 (21.11%)
NSHCPs	108 (54.27%)
SHCPs	49 (24.62%)
General characteristics, median (IQR)	
Duration (s)	137.00 (51.50, 317.00)
Likes	374.00 (99.50, 1241.50)
Saves	102.00 (39.50, 267.50)
Comments	133.00 (35.00, 489.00)
Shares	125.00 (19.00, 742.00)
Outcome scores, median (IQR) [95% CI for median]	
Completeness	4.00 (3.00, 5.00) [4.00–4.00]
GQS	3.00 (2.00, 3.00) [3.00–3.00]
mDISCERN	3.00 (3.00, 3.00) [3.00–3.00]
JAMA	3.00 (2.00, 3.00) [3.00–3.00]

*IUs* individual users, *NSHCPs* non-specialized healthcare professionals, *SHCPs* specialized healthcare professionals, *IQR* interquartile range, *CI* confidence interval

### Comparison of video characteristics between different platforms

As shown in Table 3, videos on Bilibili were significantly longer than those on TikTok (median: 315.50 s, IQR: 186.75–498.00 vs. 53.00 s, IQR: 35.00-88.50; *P* < 0.001). Bilibili videos also had higher completeness scores than TikTok videos (median: 4.00 vs. 3.00; *P* < 0.001). By contrast, TikTok videos showed higher engagement across all recorded metrics, including likes, saves, comments, and shares (all *P* ≤ 0.003).

**Table 3:** Comparison of video characteristics by platform

Variable	TikTok (*N* = 103)	Bilibili (*N* = 96)	*P* value
General characteristics, median (IQR)			
Duration (s)	53.00 (35.00, 88.50)	315.50 (186.75, 498.00)	< 0.001
Likes	714.00 (300.50, 2270.00)	150.00 (69.00, 557.75)	< 0.001
Saves	154.00 (56.00, 419.00)	74.00 (33.25, 177.50)	0.003
Comments	207.00 (76.50, 967.50)	75.50 (20.75, 230.25)	< 0.001
Shares	357.00 (101.00, 1559.00)	25.50 (6.75, 152.00)	< 0.001
Outcome scores, median (IQR) [95% CI for median]			
Completeness	3.00 (2.00, 4.00) [3.00–3.00]	4.00 (4.00, 5.00) [4.00–5.00]	< 0.001
GQS	3.00 (2.00, 3.00) [2.00–3.00]	3.00 (3.00, 3.00) [3.00–3.00]	< 0.001
mDISCERN	3.00 (2.00, 3.00) [3.00–3.00]	3.00 (3.00, 3.00) [3.00–3.00]	0.001
JAMA	3.00 (2.00, 3.00) [3.00–3.00]	3.00 (2.75, 3.00) [3.00–3.00]	0.184

*IQR* interquartile range, *CI* confidence interval

*P* values were derived from the Mann-Whitney U test

Although both platforms had median GQS and mDISCERN scores of 3.00, their score distributions differed significantly (GQS: *P* < 0.001; mDISCERN: *P* = 0.001). Bilibili showed narrower score distributions and fewer low-scoring videos than TikTok. For example, the 95% CI for the median GQS was 3.00–3.00 for Bilibili, compared with 2.00–3.00 for TikTok. Likewise, the mDISCERN IQR was 3.00–3.00 for Bilibili and 2.00–3.00 for TikTok, indicating that although the medians were the same, lower scores were more frequent on TikTok. No significant between-platform difference was observed for JAMA scores (*P* = 0.184).

### Comparison of video characteristics by uploader category

Significant differences were observed in both video duration and engagement metrics across uploader categories (Table 4). SHCPs uploaded the longest videos (median: 320.00 s, IQR: 66.00-497.00), whereas NSHCP videos received the highest engagement, with median values of 654.00 likes, 154.00 saves, 175.50 comments, and 226.00 shares (all overall *P* ≤ 0.003).

**Table 4:** Comparison of video characteristics by uploader category

Variable	IUs (*N* = 42)	NSHCPs (*N* = 108)	SHCPs (*N* = 49)	*P* value
General characteristics, median (IQR)				
Duration (s)	181.00 (94.25, 318.00)	83.00 (45.50, 202.00)	320.00 (66.00, 497.00)	< 0.001
Likes	224.00 (60.00, 740.50)	654.00 (199.00, 1934.75)	169.00 (70.00, 454.00)	< 0.001
Saves	44.50 (11.75, 202.25)	154.00 (56.00, 367.25)	75.00 (47.00, 177.00)	0.003
Comments	126.00 (38.75, 402.25)	175.50 (73.00, 730.00)	38.00 (17.00, 224.00)	< 0.001
Shares	81.50 (7.00, 292.50)	226.00 (50.75, 1036.50)	34.00 (9.00, 151.00)	< 0.001
Outcome scores, median (IQR) [95% CI for median]				
Completeness	3.00 (2.25, 4.75) [3.00–4.00]	4.00 (3.00, 4.25) [3.00–4.00]	4.00 (4.00, 5.00) [4.00–4.00]	0.018
GQS	2.00 (2.00, 2.00) [2.00–2.00]	3.00 (3.00, 3.00) [3.00–3.00]	3.00 (3.00, 3.00) [3.00–3.00]	< 0.001
mDISCERN	2.00 (2.00, 2.00) [2.00–2.00]	3.00 (3.00, 3.00) [3.00–3.00]	3.00 (3.00, 3.00) [3.00–3.00]	< 0.001
JAMA	2.00 (2.00, 2.00) [2.00–2.00]	3.00 (3.00, 3.00) [3.00–3.00]	3.00 (3.00, 3.00) [3.00–3.00]	< 0.001

*IUs* individual users, *NSHCPs* non-specialized healthcare professionals, *SHCPs* specialized healthcare professionals, *IQR* interquartile range, *CI* confidence interval

*P* values were derived from the Kruskal-Wallis test. When the overall test was significant, pairwise comparisons were performed using Dunn’s post-hoc test

**Fig. 3:**
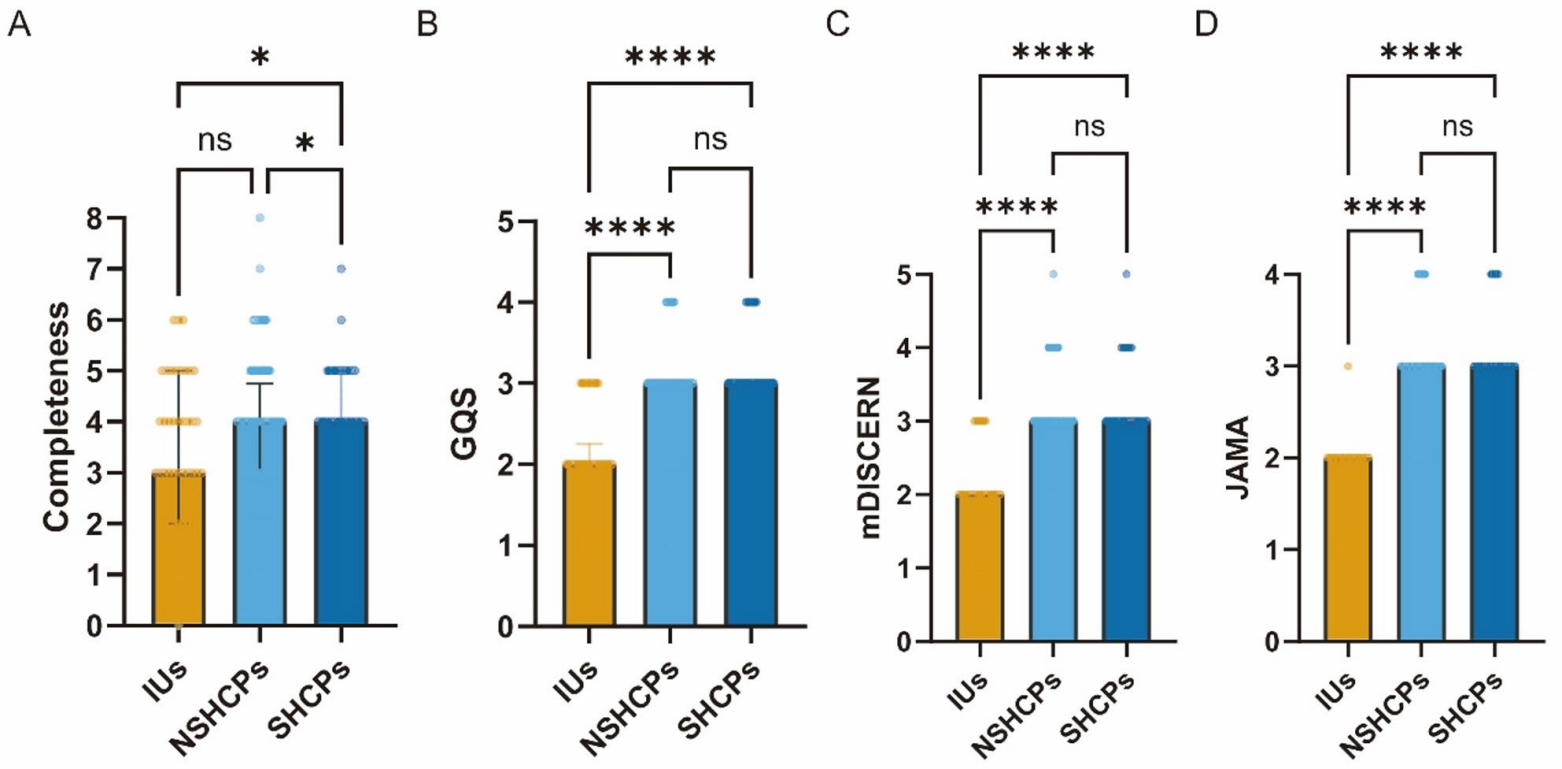
Video quality by uploader category. Comparison of content completeness and quality scores across uploader categories. (**A)** Completeness score; (**B**) Global Quality Score (GQS); (**C**) modified DISCERN (mDISCERN) score; and (**D**) Journal of the American Medical Association (JAMA) score. Data are presented as medians with interquartile ranges. Differences among groups were analyzed using the Kruskal-Wallis test followed by Dunn’s post-hoc test. *P < 0.05; ****P < 0.0001; ns, not significant. IUs, individual users; NSHCPs, non-specialized healthcare professionals; SHCPs, specialized healthcare professionals

Professional sources outperformed IUs on the established quality measures. Both SHCPs and NSHCPs had median scores of 3.00 on GQS, mDISCERN, and JAMA, whereas IUs had median scores of 2.00 across all three scales (overall *P* < 0.001 for all scales; Fig. 3B-D).

Content completeness also differed significantly among uploader groups (overall *P* = 0.018; Fig. 3A). Dunn’s post-hoc test showed that videos uploaded by SHCPs had higher completeness scores than those uploaded by IUs (*P* = 0.030) and NSHCPs (*P* = 0.048). In contrast, no significant difference was observed between videos uploaded by NSHCPs and those uploaded by IUs (*P* > 0.99).

In a supplementary descriptive analysis, videos uploaded by IUs showed the highest proportions of mentions of contraindications and complications. Contraindications were mentioned in 7.14% (3/42) of videos uploaded by IUs, 6.48% (7/108) of videos uploaded by NSHCPs, and 2.04% (1/49) of videos uploaded by SHCPs. Complications were mentioned in 78.57% (33/42), 36.11% (39/108), and 40.82% (20/49) of videos in the respective groups (Supplementary Table S4).

### Correlation analysis between video features and quality scores

**Fig. 4:**
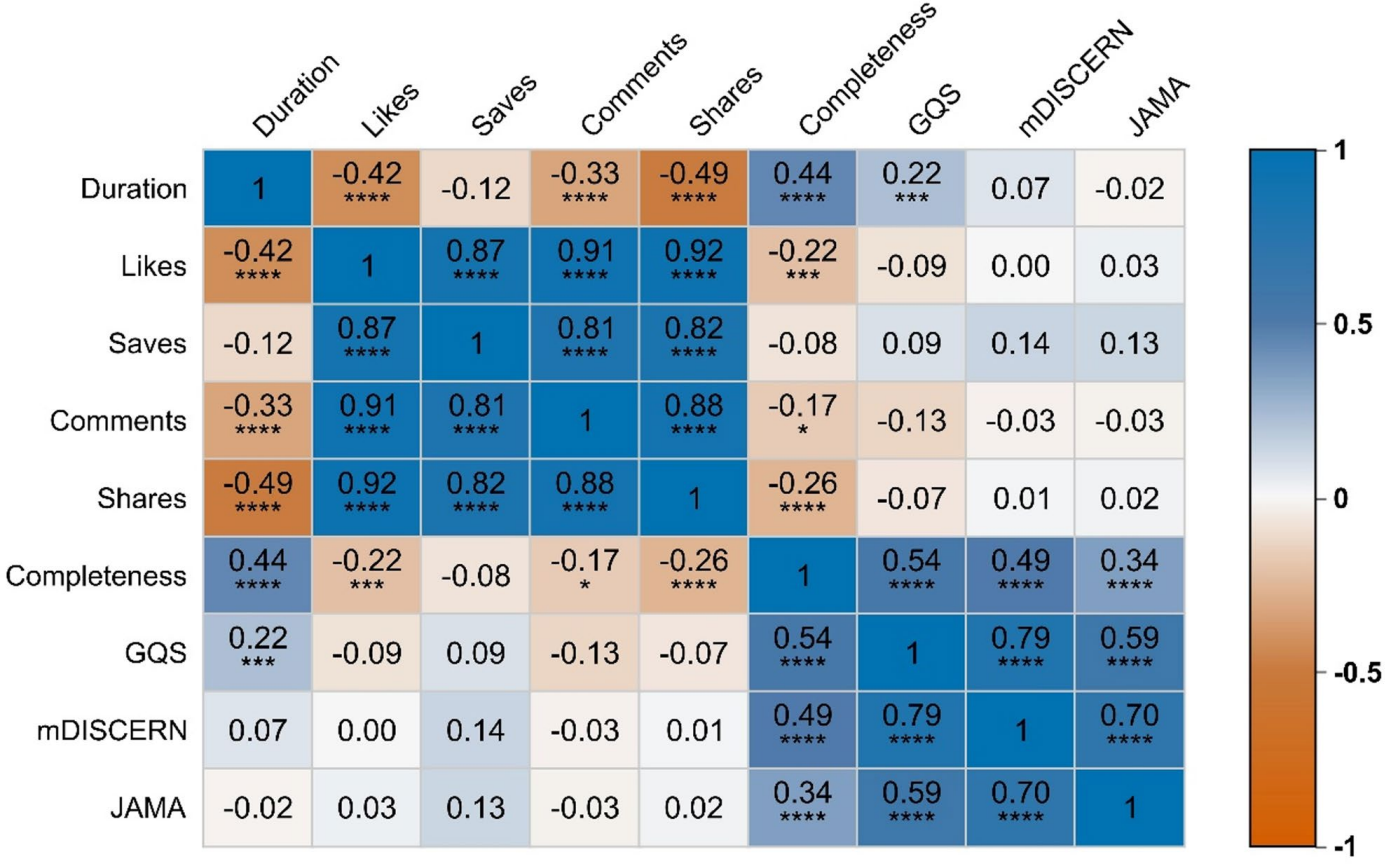
Correlations among video features and quality scores. Spearman correlation matrix showing associations among video duration, engagement metrics, content completeness, and quality scores. The color scale and numerical values represent Spearman’s rank correlation coefficients R. *P < 0.05; ***P < 0.001; ****P < 0.0001

The results of the Spearman correlation analysis are shown in Fig. 4. Video duration was moderately positively correlated with completeness (*R* = 0.44, *P* < 0.0001) and weakly positively correlated with GQS (*R* = 0.22, *P* < 0.001). By contrast, duration was negatively correlated with likes (*R* = -0.42, *P* < 0.0001), comments (*R* = -0.33, *P* < 0.0001), and shares (*R* = -0.49, *P* < 0.0001).

The engagement metrics were strongly intercorrelated, including likes with saves (*R* = 0.87), comments (*R* = 0.91), and shares (*R* = 0.92) (all *P* < 0.0001). However, these engagement indicators were not positively associated with the established quality and reliability measures. Instead, likes, comments, and shares were negatively correlated with completeness (*R* = -0.22, *P* < 0.001; *R* = -0.17, *P* < 0.05; and *R* = -0.26, *P* < 0.0001, respectively).

Substantial positive correlations were also observed among the three quality measures, particularly between GQS and mDISCERN (*R* = 0.79, *P* < 0.0001), mDISCERN and JAMA (*R* = 0.70, *P* < 0.0001), and GQS and JAMA (*R* = 0.59, *P* < 0.0001).

## Discussion

### Main findings

This study evaluated the quality, reliability, and content completeness of short videos on impacted wisdom teeth. Overall, the videos showed moderate quality and reliability. A clear pattern in content coverage was the predominance of indications and definitions, whereas contraindications and other risk-related information were seldom addressed. In addition, engagement metrics were not positively associated with the established quality measures, indicating that popularity-based platform signals are poor indicators of educational value [32–34]. Videos uploaded by professional sources, particularly SHCPs, generally performed better than those uploaded by IUs.

### Video engagement metrics

The analysis showed relatively high interaction metrics for videos related to impacted wisdom teeth, particularly those uploaded by NSHCPs. This pattern is consistent with engagement trends reported in other dental and medical settings [18, 35]. It may reflect the fact that relatability and accessibility attract viewers more readily than professional authority alone. On platforms such as TikTok and Bilibili, higher engagement may also be influenced by recommendation systems that favor content that is immediately appealing or highly relatable [36]. Accordingly, metrics such as likes, saves, comments, and shares should not be interpreted as direct indicators of educational quality.

### Content coverage and information gap

The content analysis revealed a skewed informational landscape. Crucial safety-related information, such as contraindications, appeared in only 5.53% of videos, whereas indications (76.38%) were covered extensively. This imbalance is clinically important because wisdom tooth extraction carries risks ranging from postoperative infection and dry socket to nerve injury and, in rare cases, serious systemic complications [37, 38]. Limited discussion of these risks may reduce the likelihood that viewers obtain a balanced understanding of the procedure and its implications for informed decision-making [39].

Completeness scores were also suboptimal across uploader groups. One possible explanation is that creators often adopt a serialized strategy to maintain viewer retention. Instead of producing a single comprehensive video, they divide the topic into multiple shorter clips. Although such an approach may improve platform performance, it can also fragment clinically important information.

### Comparison of video quality and reliability across different platforms

Clear structural differences were observed between the two platforms. Videos on Bilibili were significantly longer (median 315.50 s vs. 53.00 s) and had higher completeness scores (median 4.00 vs. 3.00) than those on TikTok. This pattern may reflect Bilibili’s role as a platform that accommodates longer and more detailed educational content, consistent with observations in other health communication settings [20, 21]. By contrast, TikTok showed substantially higher user engagement but lower content completeness [40, 41]. These findings suggest that platform-specific characteristics may influence how dental information is packaged and consumed, and that future health communication strategies may benefit from being adapted to the strengths and limitations of each platform.

### Differences in video quality and reliability across different uploaders

Videos uploaded by SHCPs and NSHCPs achieved significantly higher GQS, mDISCERN, and JAMA scores than those uploaded by IUs. This finding is consistent with previous studies suggesting that professionally generated dental content is generally more reliable and better structured than content produced by lay sources [42, 43].

Although IU videos achieved a median completeness score of 3.00 and often covered a relatively broad range of themes, they were more likely to contain anecdotal or potentially misleading claims. During the assessment process, recurrent examples included unsupported aesthetic claims, such as the suggestion that extracting wisdom teeth could slim the face, and exaggerated statements about severe outcomes, such as permanent memory loss. These observations were not coded using a separate misinformation scale and should therefore be interpreted as illustrative examples rather than formal prevalence estimates. Nevertheless, they suggest that broader topical coverage does not necessarily translate into more accurate or more balanced patient education.

A supplementary descriptive analysis further showed that IU videos were more likely than professionally uploaded videos to mention contraindications and, especially, complications (Supplementary Table S4). This pattern may reflect the anecdotal and experience-based nature of user-generated content, in which adverse personal experiences are more likely to be emphasized.

### Correlation between engagement metrics and quality

Strong positive intercorrelations were observed among engagement metrics, indicating that videos with high interaction tended to perform well across multiple popularity indicators. However, these metrics did not show positive associations with the established measures of quality and reliability. By contrast, some engagement indicators were negatively correlated with content completeness, indicating that user popularity should not be used as a proxy for educational quality in dental health information [44, 45].

Video duration was positively correlated with completeness (*R* = 0.44) but negatively correlated with several engagement metrics, suggesting a tension between informational breadth and platform visibility [46]. In practical terms, more comprehensive videos may offer greater educational value while being less likely to achieve strong algorithm-driven dissemination.

### Practical implications

These findings may have implications for several stakeholder groups. For viewers and patients seeking preoperative information online, popularity metrics should not be interpreted as evidence of medical accuracy, completeness, or trustworthiness. For dental professionals, the results highlight the importance of producing concise but balanced content that addresses not only indications and procedural steps, but also contraindications, complications, and postoperative expectations. For institutions and professional organizations, the findings support more active participation in short-video health communication and more explicit correction of common misconceptions. At the platform level, measures such as clearer source labeling, verified professional identification, and greater visibility for evidence-based content may help reduce exposure to incomplete or potentially misleading information. Future oral health communication strategies may therefore benefit from platform-specific content design that preserves clinical accuracy while remaining responsive to audience preferences.

### Limitations

This study has several limitations. First, the search strategy relied on a single formal Chinese clinical keyword and the top 150 comprehensively ranked videos on two Chinese platforms; therefore, videos using colloquial expressions or appearing beyond the initial search results may have been missed. Second, as a cross-sectional snapshot obtained on a single day, the study could not capture temporal changes in platform algorithms, ranking mechanisms, or content evolution. Third, only Chinese-language videos from TikTok and Bilibili were included, which limits the generalizability of the findings to multilingual audiences, other regions, and other platforms. Fourth, the IWT-CCS-8 was a study-specific checklist derived from relevant literature and clinical guidance. However, it has not been externally validated, and its equal-weight design may not fully reflect the differing clinical importance of individual domains. For example, omitting surgical contraindications may be more clinically consequential than omitting cost information. Fifth, although this study assessed content completeness, quality, and reliability, it did not conduct a statement-by-statement factual-accuracy audit of every video. Accordingly, the examples of misinformation described in the Discussion should be interpreted as illustrative observations rather than formal prevalence estimates. Sixth, user engagement metrics such as likes, comments, saves, and shares do not directly measure viewer comprehension, retention, or actual educational benefit. Finally, viewer-level demographic data were not available from the platforms, so the present study could not determine who actually watched these videos. These limitations should be considered when interpreting the findings.

## Conclusions

This cross-sectional study found that short videos on TikTok and Bilibili about impacted wisdom teeth had moderate overall quality and reliability, with limited coverage of contraindications and other risk-related information. Videos uploaded by specialized healthcare professionals generally provided more reliable and more complete information than videos uploaded by individual users, whereas engagement metrics were not useful indicators of informational quality. Given the study’s Chinese-language and platform-specific scope, these findings should be interpreted cautiously. However, they suggest that professionally produced, clinically balanced content may improve online patient education on impacted wisdom teeth. Social media videos may complement, but should not replace, professional clinical consultation.

## Supplementary Information

Supplementary Material 1: Supplementary Table S1. Global Quality Score criteria.

Supplementary Material 2: Supplementary Table S2. mDISCERN criteria.

Supplementary Material 3: Supplementary Table S3. JAMA benchmark criteria.

Supplementary Material 4: Supplementary Table S4. Mentions of contraindications and complications by uploader category.

## Data Availability

The datasets used and/or analyzed during the current study are available from the corresponding author on reasonable request.
