# Supplementary material for: Quality, reliability, and content completeness of Chinese-language short videos on impacted wisdom teeth on TikTok and Bilibili: a cross-sectional study
Source: BMC Oral Health. 2026 May 11;26:1216. doi: 10.1186/s12903-026-08553-7 (PMC13344019; doi:10.1186/s12903-026-08553-7)
Supplement: Supplementary file 1 — Supplementary Material 1: Supplementary Table S1. Global Quality Score criteria. [file 12903_2026_8553_MOESM1_ESM.docx]

**Supplementary Table S1** Global Quality Score criteria

| Score | Description |
| --- | --- |
| 1 | Poor quality; poor flow of the videos; most information missing; not at all useful for patients |
| 2 | Generally poor quality; some information listed, but many important topics missing; of very limited use to patients |
| 3 | Moderate quality; suboptimal flow; some important information adequately discussed, but other information poorly discussed; somewhat useful for patients |
| 4 | Good quality and generally good flow; most of the relevant information listed, but some topics not covered; useful for patients |
| 5 | Excellent quality and flow; very useful for patients |

GQS, Global Quality Score.
