# Supplementary material for: Quality, reliability, and content completeness of Chinese-language short videos on impacted wisdom teeth on TikTok and Bilibili: a cross-sectional study
Source: BMC Oral Health. 2026 May 11;26:1216. doi: 10.1186/s12903-026-08553-7 (PMC13344019; doi:10.1186/s12903-026-08553-7)
Supplement: Supplementary file 2 — Supplementary Material 2: Supplementary Table S2. mDISCERN criteria. [file 12903_2026_8553_MOESM2_ESM.docx]

**Supplementary Table S2** mDISCERN criteria

| Item | Criterion |
| --- | --- |
| 1 | Is the video clear, concise, and understandable? |
| 2 | Are valid sources cited? |
| 3 | Is the content presented balanced and unbiased? |
| 4 | Are additional sources of content listed for patient reference? |
| 5 | Are areas of uncertainty mentioned? |

mDISCERN, modified DISCERN.
