# Supplementary material for: Quality, reliability, and content completeness of Chinese-language short videos on impacted wisdom teeth on TikTok and Bilibili: a cross-sectional study
Source: BMC Oral Health. 2026 May 11;26:1216. doi: 10.1186/s12903-026-08553-7 (PMC13344019; doi:10.1186/s12903-026-08553-7)
Supplement: Supplementary file 3 — Supplementary Material 3: Supplementary Table S3. JAMA benchmark criteria. [file 12903_2026_8553_MOESM3_ESM.docx]

**Supplementary Table S3** JAMA benchmark criteria

| Criterion | Description | | Score |
| --- | --- | --- | --- |
| Authorship | Author and contributor credentials and their affiliations should be provided. | 1 | |
| Attribution | Clearly lists all copyright information and states references and sources for content. | 1 | |
| Currency | Initial date of posted content and subsequent updates to content should be provided. | 1 | |
| Disclosure | Conflicts of interest, funding, sponsorship, advertising, support, and video ownership should be fully disclosed. | 1 | |

JAMA, Journal of the American Medical Association.
