# Supplementary material for: Quality, reliability, and content completeness of Chinese-language short videos on impacted wisdom teeth on TikTok and Bilibili: a cross-sectional study
Source: BMC Oral Health. 2026 May 11;26:1216. doi: 10.1186/s12903-026-08553-7 (PMC13344019; doi:10.1186/s12903-026-08553-7)
Supplement: Supplementary file 4 — Supplementary Material 4: Supplementary Table S4. Mentions of contraindications and complications by uploader category. [file 12903_2026_8553_MOESM4_ESM.docx]

**Supplementary Table S4** Mentions of contraindications and complications by uploader category

| **Uploader category** | **Videos, N** | **Contraindications, N (%)** | **Complications, N (%)** |
| --- | --- | --- | --- |
| IUs | 42 | 3 (7.14%) | 33 (78.57%) |
| NSHCPs | 108 | 7 (6.48%) | 39 (36.11%) |
| SHCPs | 49 | 1 (2.04%) | 20 (40.82%) |
| Total | 199 | 11 (5.53%) | 92 (46.23%) |

Values are n (%). IUs, individual users; NSHCPs, non-specialized healthcare professionals; SHCPs, specialized healthcare professionals.
